# Torque teno virus as a marker of immune status in immunocompromised patients: A systematic review

**DOI:** 10.1111/eci.70068

**Published:** 2025-05-15

**Authors:** Janaina B. Medina, Fábio França Vieira e Silva, Rafael Antônio Velôso Caixeta, Bruna de Oliveira Rech, Alba Perez‐Jardón, María Elena Padín‐Iruegas, Mario Pérez‐Sayáns, Paulo Henrique Braz‐Silva, Karem L. Ortega

**Affiliations:** ^1^ Oral Medicine, Oral Surgery and Implantology Unit (MedOralRes), Faculty of Medicine and Dentistry University of Santiago de Compostela Santiago de Compostela Spain; ^2^ ORALRES Group Health Research Institute of Santiago de Compostela (FIDIS) Santiago de Compostela Spain; ^3^ Department of Stomatology, School of Dentistry University of São Paulo São Paulo Brazil; ^4^ Human Anatomy Area University of Vigo Vigo Spain; ^5^ Laboratory of Virology Institute of Tropical Medicine, School of Medicine, University of São Paulo São Paulo Brazil

**Keywords:** biomarker, immunosuppression, prognosis, torque teno virus, transplantation

## Abstract

**Background:**

Torque teno virus (TTV) is not known to cause disease in humans; however, chronic inflammatory conditions and immunosuppression states can favour TTV replication. This study aimed to verify the effectiveness of TTV as an immune biomarker.

**Methods:**

The protocol of this review was registered in PROSPERO (CRD42022331049) and performed according to the Preferred Reporting Items for Systematic Reviews and Meta‐Analyses guidelines.

**Results:**

Thirty‐three articles were selected and different groups of patients were assessed. In the solid organ and hematopoietic stem cell transplant groups, most studies reported that TTV viral load (VL) was highly detectable after transplantation and compared to controls, but the association with immune parameters showed conflicting results. In melanoma patients, no statistical difference in TTV VL was identified between susceptible and treatment‐resistant patients. In lung cancer patients, viral load increases significantly with disease progression but decreases after chemotherapy. HIV‐positive patients showed a higher VL than controls, but an inverse correlation with CD4+ was observed in half of the studies. Although 57.14% of all studies presented a low risk of bias, significant differences were observed between studies, particularly in the choice of the analyzed outcome, the parameter used to evaluate the patient's immune status, the presence of a control group, and the sample collection time points.

**Conclusions:**

Although TTV seems to have the potential to be a promising biomarker of immunosuppression, further high‐quality prospective clinical studies are still needed.

## Graphical abstract

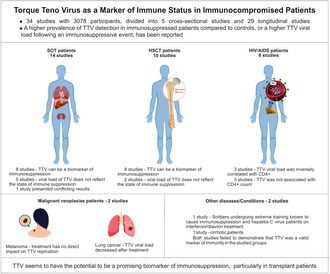

## 1 INTRODUCTION

The Anelloviridae family comprises 146 species of non‐enveloped single‐stranded circular DNA viruses, among them the torque teno virus (TTV), also known as Alphatorquevirus.^1^ TTV was first discovered in humans in 1997 in a patient with post‐transfusion hepatitis and constitutes a large fraction of the human blood virome.^2^

TTV is considered a ubiquitous virus, and although its prevalence in healthy individuals is estimated between 30% and 95%,^3^, ^4^, ^5^, ^6^ it is not known to cause any disease in humans.^7^, ^8^, ^9^

The TTV viral loads have been investigated in several situations. In patients with hepatitis B and C viruses, co‐infection with TTV is commonly diagnosed, despite not being related to the loss of liver function or the natural course of chronic hepatitis B or C.^10^, ^11^, ^12^ Higher levels of TTV viremia are correlated with lower levels of CD4+ T cells in HIV‐positive patients.^13^ There have been attempts to link TTV viral load to the level of immunosuppressive drugs in organ transplant patients and to the immune cell type most affected by the immunosuppression.^14^ Recent studies in transplant patients have shown that, in patients with high levels of immunosuppression, the risks of acute cellular rejection and antibody‐mediated rejection increase.^15^, ^16^, ^17^

The development of a technique or biomarker to identify and stage the degree of immunosuppression in patients with severe immunological deficiencies is important not only to prevent opportunistic infections and decrease morbidity and mortality of patients but also to optimise the levels of immunosuppressive drugs used in some treatments.^18^, ^19^, ^20^, ^21^, ^22^

This systematic review sought to provide an overview of the current literature on the efficacy of TTV as a potential biomarker of immunity.

## 2 MATERIALS AND METHODS

The protocol of this systematic review was previously designed by BOR and KLO, agreed upon by all authors, registered in PROSPERO (CRD42022331049), and performed according to the guidelines set by the Preferred Reporting Items for Systematic Reviews and Meta‐Analyses (PRISMA).^23^

The search question was formulated according to the PECO framework, which reads as follows: ‘Can torque teno virus be used as a marker of immune efficacy in immunosuppressed patients?’ Additionally, the PECO method involves the following: population (patients with conditions causing immune suppression), exposure (immune suppression/immune recovery), comparison (pre‐ and post‐immune suppression/immune recovery events or clinical/laboratory findings characterizing immune suppression/immune recovery) and outcome (changes in TTV viral load).

### 2.1 Search strategy

An electronic search was performed using the following databases: Cochrane, LILACS, PubMed, Scopus and Web of Science. Additionally, a gray literature search was performed using Google Scholar, ProQuest and Open Grey. The Google Scholar search was limited to the first 100 published articles, and the reference lists of the selected articles were also searched. The search was performed on January 10, 2024, and combined thesaurus terms used by them (e.g. MeSH and EMTREE) and free text words. For Medline, the following algorithm was used: (‘torque teno virus’[MeSH] OR ‘torque teno virus’ OR ‘Torque teno viruses’ OR ‘Alphatorquevirus’ OR ‘Transfusion Transmitted Virus’ OR ‘Transfusion Transmitted Viruses’ OR ‘Transfusion Transmitted Viral’ OR ‘TT Virus’ OR ‘TT Viruses’ OR ‘TTV’ OR ‘TT V’) AND (‘Immunocompromised Host’ [MeSH] OR immunity OR immune OR immuno* OR immunosuppress* OR immunocomprom* OR Immunodefic*). Syntax was adapted to each database.

### 2.2 Eligibility criteria

An ad hoc review team was formed to conduct this systematic review, in which articles were selected in three phases. In the first phase, a reference manager software (EndNote® X9 Thomson Reuters, Philadelphia, PA) was used to collect references and exclude duplicates. In the second phase, two reviewers (BOR and RAVC) independently reviewed the titles and abstracts of all the articles selected in the search. Articles that did not clearly meet the inclusion criteria or that met any of the exclusion criteria were excluded. In cases of doubt, a third reviewer (KLO) was consulted. In the third phase, the remaining articles were read in full and evaluated using the same eligibility criteria.

The inclusion criteria were as follows: (i) original research study with no language limitation and (ii) evaluation of TTV as an immunological marker in patients with immune system dysregulation (immunocompromised patients, either immunosuppressed or immunodeficient).

The exclusion criteria were as follows: (i) reviews, letters, personal opinions, book chapters, case reports or conference abstracts; (ii) animal and in vitro studies; and (iii) studies in which the patients did not present any degree of immune system dysregulation; (iv) studies that did not present assessments of TTV viral load at least at two different time points, one before and one after an event that characterized a change in the patient's immune response (such as transplantation, immunossupressive therapy, chemotherapy, antiretroviral therapy, HIV/AIDS, malignant neoplasias, chronic diseases that affects immune system and opportunistic infections), or, in the case of presenting only one TTV viral load count, did not correlate it with any type of patients' immunological marker.

### 2.3 Data extraction

Initially, two authors (FFVS and JBM) collected the necessary information from the selected articles soon after the third author (RAVC) confirmed the integrity of the information. The following data were recorded for each of the selected articles: author, year, country, study design, sample size, patient condition, control group, study objectives, fluid evaluated, TTV quantification technique, TTV‐positive patients, mean TTV viral load in Log10 DNA copies/mL, sample collection time‐points, immune parameters evaluated and conclusions. We also collected the immunosuppressive regimen of transplanted patients (SOT and HSCT), and the data are available in the Appendix S1.

### 2.4 Assessment of the risk of bias

Two reviewers (JBM and KLO) independently assessed the risk of bias in the included studies using the Joanna Briggs Institute's Critical Appraisal Checklist.^24^ Risk of bias was categorized as ‘high’ when the study reached up to 49% score ‘yes’; ‘unclear’ when the study reached 50% to 69% score ‘yes’; and ‘low’ when the study reached more than 70% score ‘yes’. The results are shown in traffic light and weighted bar graphs generated by using the generic dataset model of the Risk of Bias Visualization (ROBVIS) package.^25^

## 3 RESULTS

### 3.1 Study selection

**FIGURE 1:**
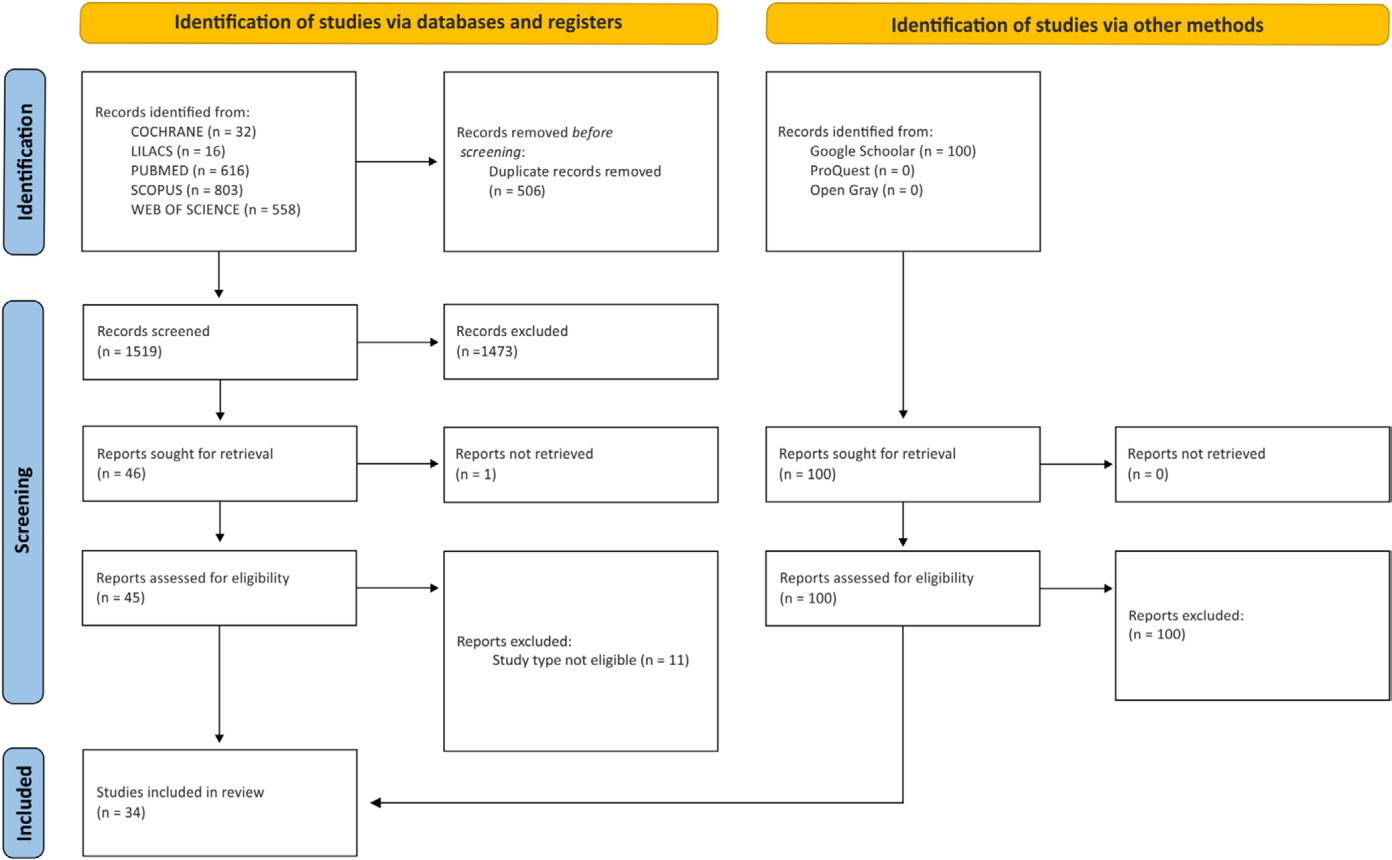
PRISMA 2020 flowchart for the systematic review.

During the initial search, 2025 citations were identified in the databases. After automatically eliminating duplicate studies by using the EndNote® X9 software, 1252 remained (Thomson Reuters, Philadelphia, USA). After reading the titles and abstracts, 46 articles were considered potentially useful and were selected for evaluation in phase 2, with 11 being subsequently excluded, and one could not be retrieved. Therefore, 34 articles were selected for full reading and data collection to answer the research questions (Figure 1).

### 3.2 Characteristics of the studies and data extraction

By analyzing the selected studies, it was possible to verify a total of 3078 participants, divided into five cross‐sectional studies^13^, ^26^, ^27^, ^28^, ^29^ and 29 longitudinal studies. Longitudinal studies were classified as such solely because they provided observations of the studied populations at least at two different time points. However, none of them can be classified as a cohort study.^2^, ^4^, ^8^, ^17^, ^30^, ^31^, ^32^, ^33^, ^34^, ^35^, ^36^, ^37^, ^38^, ^39^, ^40^, ^41^, ^42^, ^43^, ^44^, ^45^, ^46^, ^47^, ^48^, ^49^, ^50^, ^51^, ^52^, ^53^, ^54^

The total sample size ranged from 15 to 324.^8^, ^28^ Fourteen studies were carried out on solid organ transplant (SOT) recipients (kidney, liver, heart and pancreas),^2^, ^4^, ^17^, ^30^, ^33^, ^34^, ^37^, ^42^, ^45^, ^46^, ^50^, ^51^, ^52^, ^53^ 11 on hematopoietic stem cell transplant (HSCT) recipients+,^27^, ^31^, ^32^, ^35^, ^36^, ^39^, ^40^, ^41^, ^47^, ^48^, ^54^ six on HIV+ patients,^8^, ^9^, ^13^, ^18^, ^26^, ^28^, ^41^, ^43^, ^44^, ^49^, ^55^, ^56^ two on patients with cancer (lung cancer, melanoma)^38^, ^43^ and one in patients with cirrhosis^29^ (Table 1).

**TABLE 1:** Characteristics of the selected studies about TTV.

Author, year; country	Study design and sample size	Patients condition	Control group	Study objectives	Fluid evaluated	TTV quantification technique (method, kit and gene region)	TTV positive patients	Sample collection time‐points	Mean TTV VL—Log10 DNA copies/ml (Range)	Immune parameters evaluated	Conclusions
HIV											
Shibayama et al., 2001; Japan	Cross sectional *N* = 144	HIV+ (treatment‐naive)	BD (age and sex‐matched)	Prevalence and TTV VL and its relationship with CD4+	Serum	qPCR UTR PCR and N22 qPCR In‐house UTR and N22 Detection limit = NI	UTR: HIV = 99%; BD = 91% N22: HIV = 56%; BD = 27%	One time before starting the treatment	UTR: HIV = 4.2 ± 1.2 BD = 3.1 ± 0.9 N22: HIV = 2.6 ± 1.5 BD = 1.5 ± 0.9	UTR: CD4+ <100 TTV VL = 5.2 ± 1.5; CD4+ >100 TTV VL = 3.2 ± 1.7 N22: CD4 + <100 TTV VL = 3.2 ± 1.7; CD4+ >100 TTV VL = 2.3 ± 1.4	TTV is highly prevalent and high‐titred in HIV+ and TTV viral load is inversely correlated with CD4 + .
Madsen et al., 2002; Denmark	Longitudinal *N* = 15	HIV+ with impaired immune status	BD (*N* = 62)	Correlation between TTV VL and functional immune reconstitution	Plasma	Competitive PCR assay In‐house GG‐rich region (UTR) Detection limit = 875 copies/mL	HIV before HAART = 100% BD = 42% HIV after HAART = NI	Before HAART: 1st = 307d 2nd = 120d 3rd = 0d After HAART: 4th = 63d 5th = 196d 6th = 357d	Before HAART = 5.73 1 year after HAART = 4.78 BD = NI	CD4+	TTV VL reduction after HAART. Increase in CD4+ after HAART was not statistically significant.
Shieh et al., 2003; Taiwan	Cross sectional *N* = 62	HIV+ (treatment‐naive)	Healthy (*N* = 105)	Correlate the prevalence of nine viruses (including TTV) with CD4+ cell counts and compared with healthy controls	Plasma	Double‐nested PCR In‐house UTR and N22 Detection limit = NI	HIV = 32% Healthy = NI	One time. Before taken any ART	HIV = NI Healthy = NI	CD4+ In TTV positive CD4+ 191; in TTV negative CD4+ 295	TTV detection was significant higher in HIV+ patients but was not associated with CD4+ count.
Schmidt et al., 2021; Germany	Longitudinal *N* = 301	HIV+	No	TTV VL to predict immune recovery during cART.	Plasma	RT‐PCR In‐house UTR Detection limit = 1000 copies/mL	Before cART = 96% After cART = NI	Before cART After cART (within the first year)	Before cART = 5.37 Before cART: CD4 < 100 TTV VL = 6.23; CD4 100–200 TTV VL = 5.71; CD4 >200 TTV VL = 5.17 After cART (recovery): Gain CD4+ <50 TTV VL = 5.68; Gain CD4+ 50–200 TTV VL = 5.44; Gain CD4+ >200 TTV VL = 4.99	CD4	TTV VL is inverse correlated with CD4+ at baseline and after cART.
Honorato et al., 2022; Brazil	Cross sectional *N* = 276 HIV+ on ART; 48 AIDS‐KS on CT	HIV+	No	TTV as a potential biomarker of immune status in HIV+ patients	Saliva	RT‐PCR In‐house UTR Detection limit = 40 copies/mL	HIV+ ART = 80% AIDS‐KS = 87%	One time	HIV+ ART = 3.1 AIDS‐KS = 5.3 CD4+ <200 TTV VL = 5.7 CD4+ 201–350 TTV VL = 3.7 CD4+ 351–500 TTVL = 3.4 CD4 >500 TTV VL = 2.9	CD4	TTV VL was inversely correlated with the CD4+.
Abbate et al., 2023; Italy	Longitudinal *N* = 21	Acute HIV infection (AHI)	Healthy donors (*N* = 13) and chronically HIV + with CD4+ <200 or AIDS and ART‐naïve (*N* = 28)	TTV VL as a marker of immune reconstitution	PBMC	RT‐PCR In‐house UTR Detection limit = NI	T0 = 100% T1 = NI T2 = NI	T0 = at serodiagnosis T1 = 3 months after ART T2 = 12 months after ART	T0 = 3.65 log copies/10^6^ PBMC; T1and T2 NI	CD4 and CD8 differentiation, activation, exhaustion, and senescence phenotypes	TTV was higher in AHI than in healthy controls and lower than in HIV‐ chronically infected. TTV loads were unrelated to CD4+ counts. TTV loads were positively correlated with the CD8+CD57+ cells.
Solid organ transplant											
Moen et al., 2003; Norway	Longitudinal N = 10	KTx	No	Effect of immunosuppressants on TTV VL	Serum	RT‐PCR In‐house ORF2 Detection limit = NI	Before IT = 100% After = NI	Before IT and 2.5 to 13 months after surgery	NI	None	Increases in TTV VL, particularly during the first 50 days after the transplantation.
Focosi et al., 2015; Italy	Longitudinal *N* = 70	KTx and/or PTx	No	Effect of the induction regimens of immunosuppression (anti‐thymocyte globulins (ATG) or basiliximab) on TTV VL	Plasma	RT‐PCR In‐house UTR Detection limit = 100 copies/mL	NI	Before induction Post Tx: +7 +15 +30	NI	T‐lymphocyte counts and regimens of immunosuppression	ATG induced higher drops in TTV VL than basiliximab Decline in TTV VL, in parallel with elimination of peripheral blood lymphocytes.
Görzer et al, 2015; Austria	Longitudinal *N* = 46	LTx	No	TTV VL dynamics in LTx	Plasma	RT‐PCR In‐house UTR Detection limit = 100 copies/mL Linear range = 100 copies/mL to 10 log_10_ copies/mL	Pre Tx = 93% Post Tx = 100%	Pre Tx Post Tx (up to 3 months)	Pre Tx = 4.4 Post Tx = 7.0	None	TTV VL is higher after using immunosuppressant drugs.
Jaksch et al., 2018; Austria	Longitudinal *N* = 143	LTx	No	Levels and kinetics of TTV after LTx and relation to the development of acute and chronic rejection, infectious complications and as a marker of immunosuppression	Plasma	RT‐PCR In‐house UTR Detection limit = 100 copies/mL	Pre Tx = 82% Post Tx = 100%	Between 2 weeks and 2 months	Pre Tx = 3.6 Post Tx (134 ± 87 days) = 9.5	Opportunistic infections, acute cellular rejection, and tacrolimus level	TTV VL increased after transplant. TTV VL correlates with levels of tacrolimus.
Maggi et al., 2018; Italy	Longitudinal *N* = 280	KTx or LiTx	BD (30)	TTV kinetics in SOT recipients and the relationship with transplanted organ, IT and CMV reactivationpost Tx	Plasma	RT‐PCR In‐house UTR Detection limit = 10 copies/mL	Pre Tx = 92% Post Tx = 100% BD = 68%	Pre Tx Post Tx: +10 +20 +30 +40 +50 +60 +70 +80 +90 +120 +180 +365	Pre Tx: KTx = 3.9 LiTx = 4.2 Post Tx KT: +10 = 3.5 +20 = 3.5 +30 = 5.0 +40 = 5.2 +50 = 5.4 +60 = 5.3 +70 = 5.5 +80 = 6.5 +90 = 6.9 +120 = 5.7 +180 = 6.9 +365 = 5.7 Post Tx Li: +10 = 4.0 +20 = 4.3 +30 = 4.7 +40 = 5.1 +50 = 6.0 +60 = 6.1 +70 = 6.0 +80 = 6.5 +90 = 6.1 +120 = 6.0 +180 = 6.4 +365 = 6.3	CMV Reactivation (Opportunistic infection)	TTV viremia could be used in early post‐transplantation period to predict CMV reactivation.
Nordén et al., 2018; Sweden	Longitudinal *N* = 98	LTx	No	TTV and EBV levels in relation to the frequency of infectious events and acute rejections	Serum	Droplet Digital PCR In‐house Region NI Detection limit = NI	NI	Post Tx: +1 month +2 months +3 months +4.5 months +6 months +9 months +12 months +18 months +24 months	Post Tx: 1–3 months = 6.7 3–6 months = 6.4 6–12 months = 5.8 12–24 months = 5.2	Opportunistic infections and acute graft rejection	No statistically significant association was found between TTV VL and opportunistic infections or acute rejection event. TTV VL did not reflects the state of immune suppression.
Fernández‐Ruiz et al., 2019; Spain	Longitudinal *N* = 221	KTx	No	TTV kinetics to predict complications of excessive immunosuppression (opportunistic infection or de novo malignancy)	Plasma	RT‐PCR Commercial (TTV R‐GENE® kit) UTR Detection limit = 167 copies/mL Linear range = 2.1 × 10^2^ to 2.1 × 10^7^ copies/mL	Pre Tx = 97.6% Post Tx = NI	Pre Tx (9 days before) Post Tx: +7 day +1 month +3 months +6 months +12 months	Pre Tx = 2.9 Post Tx: +90 = 5.0 + 180 = 5.7	CD3+, CD4+ and CD8+, infections and graft rejection	Significant inverse correlations between TTV VL and CD3+, CD4+ and CD8+ T cell counts (since month 1 and more evident by month 3). TTV DNA kinetics identify KTx recipients at increased risk of immunosuppression‐related complications
Uhl et al., 2020; Austria	Longitudinal *N* = 45	KTx‐children	No	TTV VL and its changes	Plasma	RT‐PCR In‐house UTR Detection limit = 100 copies/mL Linear range = 100 to 1 × 10^9^ copies/mL	KTx children = 100%	Once monthly for a period of 1 year	KTx children = 5.8	Doses of IT, and opportunistic infections.	TTV load positively correlated with the dose of prednisolone and mycophenolate mofetil. TTV VL was not correlate with the dose of tacrolimus, rapamycin, cyclosporine. TTV VL was not associated with infection
Batista et al., 2022; Brazil	Longitudinal *N* = 71	KTx	No	Correlation between TTV VL and immune function	Saliva and Plasma	RT‐PCR In‐house UTR Detection limit = NI	Pre Tx: Saliva = 58% Plasma = 60% Post Tx +20: Saliva = 52% Plasma = 73% Post Tx +60: Saliva = 60% Plasma = 90%	Pre Tx (24 hours) Post Tx: +20 +60	Pre Tx: Saliva = 3.40 Plasma = 3.85 Post Tx +20: Saliva = 4.12 Plasma = 3,40 Post Tx +60: Saliva = 5.04 Plasma = 7.99	None	TTV VL is higher after transplantation.
Berg et al., 2023; Denmark	Longitudinal *N* = 106	HTx	No	Kinetics of TTV to evaluate its potential as a biomarker of immunosuppression	Serum or plasma	RT‐PCR In‐house UTR Detection limit = 12 × 10^3^ copies/mL	T0 = 98.11% T1 = NI T2 – NI T3 = NI T4 = NI T5 = NI T6 = NI	Post Tx: T0 = 1 week T1 = 1 month T2 = 2 months T3 = 3 months T4 = 6 months T5 = 12 months T6 = 36 months	Post Tx: T0 = 4.39 T1 = 6.26 T2 = 7.98 T3 = 8.23 T4 = 7.94 T5 = 7.55 T6 = 5.49	Tacrolimus serum concentration, B cells, CD4 + and CD8+	TTV VL was not associated with immunosuppression (tacrolimus, B cells, CD4+ and CD8+)
Benning et al., 2023; Germany	Longitudinal *N* = 43	KTx	Hemodialysis (18) Healthy controls (18)	Effect of MPA withdrawal to changes in TTV VL in SARS‐CoV‐2 vaccinated patients	Serum	RT‐PCR Commercial (TTV R‐GENE® kit) UTR Detection limit = 250 Linear range = 250 to 10^9^ copies/mL	T0 = 100% T1 = NI T2 = NI	T0 = before MPA withdrawal T1 = At time of reintroduction of MPA T2 = Two months after reintroduction of MPA	T0 = 4.1 T1 = 3.7 T2 = 4.3	Specific SARS‐CoV‐2 Anti S1‐IgG	TTV VL reflects short‐term changes in immunosuppressive therapy in KTxs in whom MPA was withdrawn to increase SARSCoV‐2 vaccine immunogenicity.
Querido et al., 2023; Portugal	Longitudinal *N* = 81	KTx	No	TTV VL kinetics, before and after KTx	Plasma	RT‐PCR Commercial (TTV R‐GENE® kit) UTR Detection limit = 100 copies/mL	Pre Tx = 72,5% Post Tx = 97.53%	Pre Tx Post Tx: +1 week +1 month +3 months +6 months +9 months +12 months	Pre Tx = 3.10 Post Tx: +3 months (peak) = 7.2 + 6 months = 6.1	Infectious events, immunoglobulins, complement, lymphocyte absolute counts and subsets	A progressive increase in TTV viral load was observed from baseline to peak at month 3 and a slight decrease was seen after month 6. TTV VL was not correlated with immunological parameters.
Mafi et al., 2023; France	Longitudinal *N* = 64	KTx	No	TTV VL and QuantiFERON®‐CMV assay to predict CMV reactivation	Plasma	RT‐PCR Commercial (TTV R‐GENE® kit) UTR Detection limit = 250 copies/mL Linear range = 250 copies/mL to 1 × 10^9^ copies/mL	NI	Pre Tx = D0 Post Tx: M1 = 1 month M2 = 2 months M3 = 3 months M4 = 4 months M5 = 5 months M6 = 6 months M8 = 8 months M10 = 10 months M12 = 12 months	D0 = 2.6 M3 = 6.5 M12 = 4.5	CMV serostatus, CMV VL and QuantiFERON®‐CMV	TTV VL did not predict CMV reactivation but rather identified patients at low risk of CMV reactivation
Cañamero et al., 2023; Spain	Longitudinal *N* = 54	KTx	No	TTV VL and its association with exposure to mycophenolate mofetil and tacrolimus	Plasma	RT‐PCR In‐house UTR Detection limit = 1 × 10^3^ copies/mL	Pre Tx = 100% Post Tx = NI	Pre Tx Pos Tx: +30 +90	Pre Tx = 2.00 Post Tx: +30 = 2.81 +90 = 6.73	Acute rejection, opportunistic infection, levels of tacrolimus, thymoglobulin and MPA	TTV VL did not relate to mean tacrolimus and MPA blood levels. TTV VL at one and third months post Tx was related to the risk of infection. TTV VL was related to induction but was not related to overall exposure to maintenance immunosuppression
Hematopoietic stem cell transplantation											
Focosi et al., 2010; Italy	Longitudinal *N* = 50	HSCT	No	TTV VL and immune system recover	Plasma	RT‐PCR In‐house UTR Detection limit = 100 copies/mL	Pre Tx = 92% Post Tx = NI	Pré Tx Post Tx: +40 +70 +100	Pre Tx = (3.1 to 7.8) Post Tx = average increase of approximately 1.5 at +100	Immunophenotyping of peripheral blood cells (CD45, CD3, CD4, CD8, CD16, CD25, CD56 and CD57)	TTV VL increased after IT. TTV viremia correlated positively only with CD8 + 57+ T lymphocytes (at baseline and at day +100) and with the dose of melphalan
Zanotta et al., 2015; Italy	Longitudinal *N* = 27	HSCT—children	Children (*N* = 22)	Cytokines in relation to clinical course of HSCT and TTV infection	Plasma	RT‐PCR In‐house UTR and ORF1 (genotypes) Detection limit = NI	Pre Tx = 40.74% Post Tx = 59.26% Total (pre + post Tx) = 81.4% Controls = NI	Pre Tx Post Tx (up to 605 days)	Pre Tx = (5.49 to 9.36) Post Tx (peak) = 6.38 Controls = NI	Inflammatory profile (cytokines)	IFN‐γ, TNF‐α, FGF‐basic and MCP‐3 were found up‐regulated and significantly associated to TTV.
Gilles et al., 2017; Germany	Longitudinal *N* = 23	HSCT Low risk group (LR) – without aGVHD (*N* = 12) High risk group (HR) – with aGVHD (*n* = 11)	Healthy (*n* = 15)	Dynamics of TTV VL after HSCT related to aGVHD.	Plasma	RT‐PCR In‐house UTR Detection limit = NI	LR: +30 Tx = 66.7% +100 Tx = 100% +200 Tx = 100% HR: +30 Tx = 100% +100 Tx = 100% Healthy = 33.3%	Post Tx: +30 +100 +200	LR Post Tx: +30 = 6.4 +100 = 9.48 +200 = 7.15 HR Post Tx: +30 = 9.26 +100 = 10.15 +200 = 7.4 Healthy = 5.08	Viral infections (CMV, EBV, BKPyV), leucocyte, lymphocyte, and neutrophil counts and aGVHD	HSCT recipients affected by aGVHD showed a significantly higher median TTV VL at day +30 than patients with a less complicated clinical course. Higher TTV levels in immunocompromised patients compared to immunocompetent controls.
Wohlfarth et al., 2018; Austria	Longitudinal *N* = 50	HSCT	No	TTV plasma dynamics and clinical associations in patients following HSCT	Plasma	RT‐PCR In‐house UTR Detection limit = 100 copies/mL Linear range = 100 to 1 × 10^10^ copies/mL	Pre Tx = 80% Post Tx: +10 = 90% +50 = 100%	Pre Tx Post Tx: +10 +30 +50 +80 +120 +160 +200 +250 +300 +365	Pre Tx = 5.37 Post Tx: Peak load +79 = 8.32	Lymphocytes, GVHD and opportunistic infections	TTV VL and lymphocyte counts inversely correlated after engraftment. The use of ATG resulted in lower TTV VL at day +10 and +30 and at day +80 and + 120. Escalated immunosuppressive therapy was followed by an increase in TTV VL. Significant correlation between TTV VL and CMV and EBV VL. Several variables can be correlated with TTV levels, but their complex interactions might perturb the capability of TTV to predict immune‐related complications
Albert et al., 2019; Spain	Longitudinal *N* = 33	HSCT	No	Characterized the dynamics of TTV DNA load	Plasma	RT‐PCR In‐house UTR Detection limit = NI	NI	Pre Tx: Post Tx: + 20 +30 +40 +50 +60 +90 +120 +150 +180 +210	NI	Absolute lymphocyte counts	TTV DNA loads measured at late times after allo‐HSCT may reflect the net state of immunosuppression of patients
Mouton et al., 2020; France	Cross sectional *N* = 41	HSCT	BD (*N* = 80)	Correlation between TTV VL, immune cell counts, and lymphocyte competence	Plasma	RT‐PCR Commercial (TTV R‐GENE® kit) UTR Detection limit = NI	HSCT = 100% BD = 68%	One time. 6 months post Tx	HSCT = 3.9 BD = 2.1	CD3+, CD4+, CD8+ and opportunistic infections	TTV VL inversely correlated with T‐cell function but not with T cell count. Significantly higher TTV VL in HSCT with viral opportunistic infection/reactivation.
Peker et al., 2020; Turkey	Longitudinal *N* = 33	HSCT‐children	Volunteers without chronic disease (*N* = 38)	Presence of TTV in pediatric HSCT patients and kinetics of TTV VL in patients with infectious complications	Plasma	RT‐PCR In‐house UTR Detection limit = 100 copies/mL Linear range = 100 to 1 × 10^10^ copies/mL	HSCT = 100% Volunteers = 97.4%	Immediately post Tx 30 d after engraftment Post Tx: 31–60 d 61–100 d	Post Tx: Until the 30 day after engraftment = 5.98 31–60 days post engraftment = 8.03 Volunteers = 5.51	Viral reactivation (CMV, EBV, ADV), aGVHD and lymphocytes count	TTV VL positively and weakly correlated to lymphocyte count. TTV VL did not correlate with other virus. Significant changes in TTV virus kinetics are observed about the recovery of immune cells in the first 100 days after HSCT.
Pradier et al., 2020; Switzerland	Longitudinal *N* = 168	HSCT	BD (*N* = 91)	Kinetic of TTV and its relationship with clinical parameters and post Tx reconstitution and complications	Plasma	RT‐PCR In‐house UTR Detection limit = 25 copies/mL Linear range = 250 to 2.5 × 10^9^ copies/mL	Pre Tx = 90,0% Post Tx = NI BD = NI	Pre Tx Post Tx: +50 +100 +150 +200 +300 +400 +547 2 to 9 years	Pre Tx = 2.4 Post Tx: +100 = 6.4 + 1460 = 4 BD = 2.2	CD3+, CD4+, CD8+, NK	TTV VL at day +100 inversely correlated with CD4 and NK TTV VL at day +300 to +400 inversely correlated with CD4 and CD8 naïve subsets.
Schmitz et al., 2020; Germany	Longitudinal *N* = 123	HSCT	No	TTV VL as a predictive marker for immune‐related clinical complications	Plasma	RT‐PCR In‐house UTR Detection limit = 100 copies/mL Linear range = 100 to 10^8^ copies/mL	Pre Tx = 50.4% Post Tx = 100%	0‐15d 16‐30d 31‐45d 46‐60d 61‐80d 81‐99d 100‐119d 120‐140d 141‐160d 161‐180d 181‐200d 201‐219d 221‐239d 240‐260d 261‐280d 281‐300d 301‐320d 321‐345d	NI	Opportunistic infection (CMV, EBV, BKPyV, HSV‐1, HHV‐6), GVHD, lymphocyte (CD8+, CD3+, CD19+, CD45+, CD4+, NK)	No correlation between TTV VL and subtype of lymphocytes, other virus and GVHD. ATG dose was associated with a significantly higher TTV VL.
Spiertz et al., 2023; Germany	Longitudinal *N* = 59	HSCT	No	TTV relation with replication control of CMV, EBV and BKPyV	Blood	RT‐PCR In‐house UTR Detection limit = NI	Between days −7 and +10 = 77.7% Post Tx (1 year after) = 95.6%	Between day −7 and +10 Post Tx: +14 +21 +28 +56 +90 +365	Between day −7 and +10 = 3.14 Post Tx: +56 = 7.10 +365 = 5.92	CMV, EBV and BKPyV	TTV VL did not correlate with other virus infection or reactivation.
Forqué et al., 2023; Spain	Longitudinal *N* = 75	HSCT	No	Investigate whether quantification of TTV, VL can be related to infections and aGVHD.	Plasma	RT‐PCR In‐house UTR Detection limit = NI	Pre Tx = 77%; T0 = 78%; Post Tx: +30 = 63% +60 = 94% + 90 = 100% +120 = 96% +180 = 97%	Pre Tx Baseline = T0 Post Tx: +30 +60 +90 +120 +180	Pre Tx = 4.15 T0 = 4.41 Post Tx: +30 = 3.40 +60: 6.24 +90: 7.29 +120: 7.12 +180 = 6.53	Occurrence of aGVHD and opportunistic infections (pneumonia and BKPyV associated haemorrhagic cystitis)	TTV DNA levels were significantly higher in patients who subsequently developed BKPyV haemorrhagic cystitis (HC). TTV DNA VL at day +30 was significantly higher in patients who went on to develop aGvHD than in those who did not
Malignant neoplasias											
Sawata et al., 2018; Japan	Longitudinal *N* = 48	Lung cancer P1—Patients with partial response or stable disease P2—Patients with progressive disease	No	TTV DNA titers in primary lung cancer and the influence of IPF on changes in TTV titers.	Serum	qPCR UTR PCR and N22 qPCR In‐house UTR and N22 Detection limit = NI	Before CT = 100% After CT = NI	Before CT After second cycle of CT (between 2 and 4 weeks)	Before CT = 4.60 P1 = 4.73 P2 = 4.08 After CT: P1 = 4.20 P2 = 4.86	Response to CT and presence of IPF	TTV DNA titer is correlated with tumor growth and CT responses
Pescarmona et al., 2021; France	Longitudinal *N* = 43	Melanoma	BD (*N* = 43)	TTV replication as a biomarker of immune checkpoint inhibitors efficacy	Plasma	RT‐PCR Commercial (TTV R‐GENE® kit) UTR Detection limit = NI	Before treatment:All melanoma patients = 69.8% Patients treatment‐sensitive tumor = 61.1% Patients treatment‐resistent tumor = 76% BD = 72.1% After 6 months treatment: All melanoma patients = 60.4%	Two sample collections in the day treatment and after 6 months of treatment	Before treatment: All melanoma patients = 2.20 Treatment sensitive = 2.26 Treatment‐resistant = 2.13 BD = 1.38 After 6 months treatment: All melanoma patients = 1.88	None	TTV VL was not different in melanoma patients before anti‐PD‐1 introduction and did not allow to distinguish patients with treatment‐sensitive tumors from patients with treatment‐resistant tumor
Other diseases/conditions											
Moen et al., 2003; Norway	Longitudinal *N* = 25	Soldiers undergoing extreme training known to cause immunosuppression	No	Effect of extreme training program designed to cause both physical and mental exhaustion on TTV VL	Serum	RT‐PCR In‐house ORF2 Detection limit = NI	Day 0 = 72% Day 5 = NI	Prior to (day 0) and during (day 5) a 1 week of intensive training program	NI	None	Insignificant increases in TTV VL were observed
Moen et al., 2003; Norway	Longitudinal *N* = 9	HCV+	No	Effect of interferon/ribavirin on TTV VL	Serum	RT‐PCR In‐house ORF2 Detection limit = NI	Before treatment = 77.78% During and after interferon/ribavirin = NI	Before, during and after 6 months of interferon/ribavirin	Before interferon = 2.9 After interferon = 3.17	None	Viremia decreased during the first months of treatment but returned before the end of the treatment period in at least half of the cases to levels comparable to those prior to treatment.
Falabello de Luca et al., 2023; Brazil	Cross sectional *N* = 72	Cirrhosis	No	TTV VL of cirrhotic individuals on the transplant waiting list	Plasma and saliva	RT‐PCR In‐house UTR Detection limit = 40 copies/mL	Plasma = 38.8% Saliva = 93.0%	One time	Plasma = 2 Saliva = 2.4	Leukocyte, lymphocyte, neutrophil, MELD and cirrhosis decompensation	TTV was more frequently identified in the saliva than in the plasma of cirrhotic patients, with higher VL in the former. TTV VL did not correlated with white blood cell count, MELD and cirrhosis decompensation.

Abbreviations: aGVHD, acute graft‐versus‐host disease; ALT, alanine aminotransferase; ART, antiretroviral therapy; ATG, rabbit‐derived antithymocyte globulin; BD, blood donors; BKPyV, polyomavirus BK; cART, combined antiretroviral therapy; CD4+, CD4+ T lymphocytes; CMV, cytomegalovirus; CNI, calcineurin inhibitors; CRS, cytokine‐release syndrome; CT, chemotherapy; d, days; DNA, deoxyribonucleic acid; EBV, Epstein–Barr virus; GVHD, graft‐versus‐host disease; HBV, hepatitis B virus; HCV, hepatitis C virus; HEV, hepatitis E virus; HGV, hepatitis G virus; HHV‐6, human herpesvirus type 6; HHV‐8, human herpes virus 8; HIV, human immunodeficiency virus; HPB‐19, human parvovirus B19; HPgV‐1, human pegivirus 1; HSTC, hematopoietic stem cell transplant; HSV‐1, herpes simplex virus type 1; HTLV‐1, human t‐lymphotropic virus 1; HTx, heart transplant; ICANS, immune effector cell‐associated neurotoxicity syndrome; IPF, idiopathic pulmonary fibrosis; IR, immune reconstitution; IT, immunosuppressive therapy; JCPyV, polyomavirus virus JC (John Cunningham); KTx, kidney transplant; LiTx, liver transplant; LTx, lung transplant; MCP‐3, monocyte chemotactic protein‐3; MELD, model of end‐stage liver disease; MPA, mycophenolic acid; NI, not informed; PBMC, peripheral blood mononuclear cells; PTx, pancreas transplant; SOT, solid organ transplant; SOT, solid organ transplant; TAV, total *Anelloviridae*; TLMV, TTV‐like‐mini‐virus; TTV, torque teno virus; Tx, transplant; VL, viral load.

The immunosuppression of the patients was assessed using the CD4+ count,^8^, ^13^, ^26^, ^27^, ^28^, ^44^ immunophenotyping of peripheral blood cells,^32^ lymphocyte counts (total, types and subtypes).^2^, ^4^, ^29^, ^31^, ^32^, ^39^, ^40^, ^41^, ^46^, ^49^, ^51^, ^54^ Some authors evaluated the immunological status of the patients indirectly due to the presence of opportunistic infections,^2^, ^17^, ^27^, ^30^, ^31^, ^37^, ^39^, ^41^, ^42^, ^44^, ^48^, ^51^, ^53^ levels or response to immunosuppressive therapy,^30^, ^33^, ^38^, ^42^, ^46^, ^53^ presence of other virus,^17^, ^28^, ^36^, ^47^, ^51^, ^52^ production of specific severe acute respiratory syndrome coronavirus 2 (SARS‐CoV‐2) vaccine response^50^ and inflammatory profile.^35^ Other authors, assuming that certain diseases are widely known to cause immunosuppression, sought to identify the dynamics of TTV viral load in this context^34^, ^43^, ^45^ and to identify the correlation between TTV viral load and conditions that can occur after significant drug immunosuppression, such as graft rejection^2^, ^30^, ^31^, ^36^, ^37^, ^46^, ^53^ and graft‐versus‐host disease (GVHD)^48^ (Table 1).

### 3.3 Quality assessment of the articles

**FIGURE 2:**
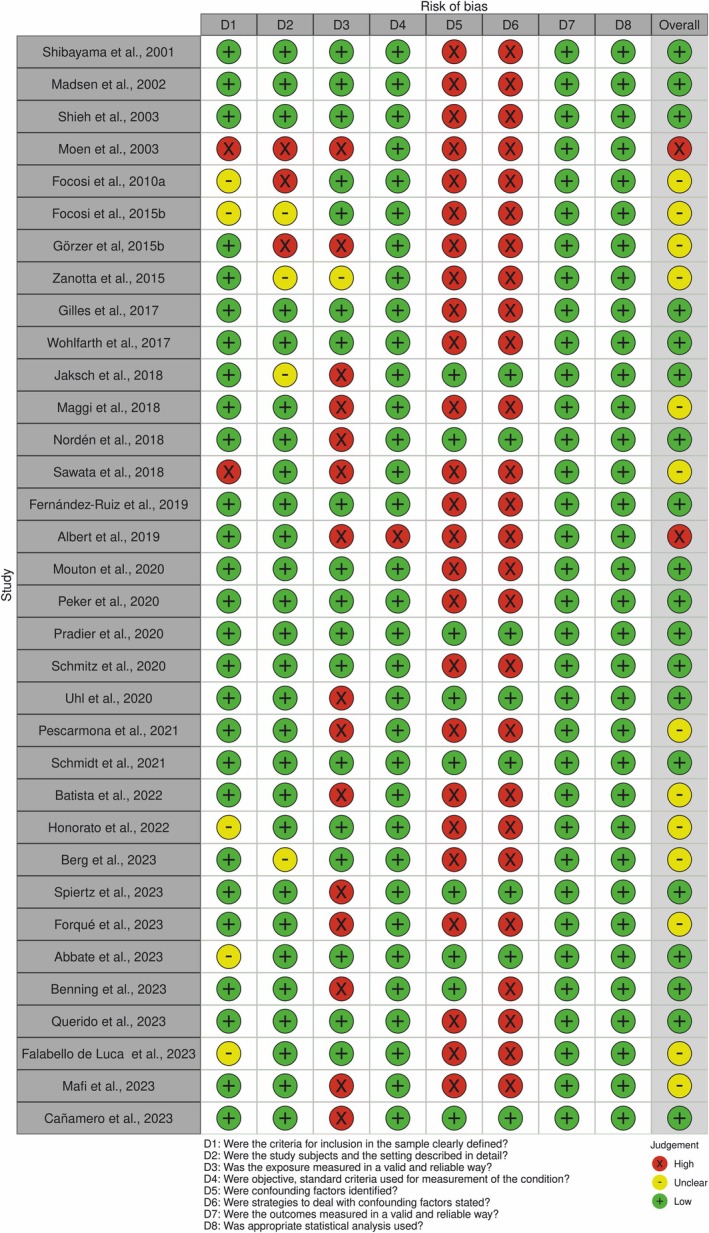
Traffic light plots of the domain‐level judgements for each individual result.

The risk of bias analysis showed that most of the studies included in this systematic review had a low risk of bias, that is 57.58%.^2^, ^8^, ^13^, ^26^, ^27^, ^30^, ^31^, ^36^, ^37^, ^39^, ^40^, ^41^, ^42^, ^44^, ^47^, ^49^, ^50^, ^51^, ^53^ However, 39.40%^4^, ^17^, ^28^, ^29^, ^32^, ^34^, ^35^, ^38^, ^43^, ^45^, ^46^, ^48^, ^52^, ^54^ were considered to have an unclear risk of bias, which is also considered a comparatively high number. In 3.03%^33^ of the studies, the risk of bias was considered high (Figure 2).

**FIGURE 3:**
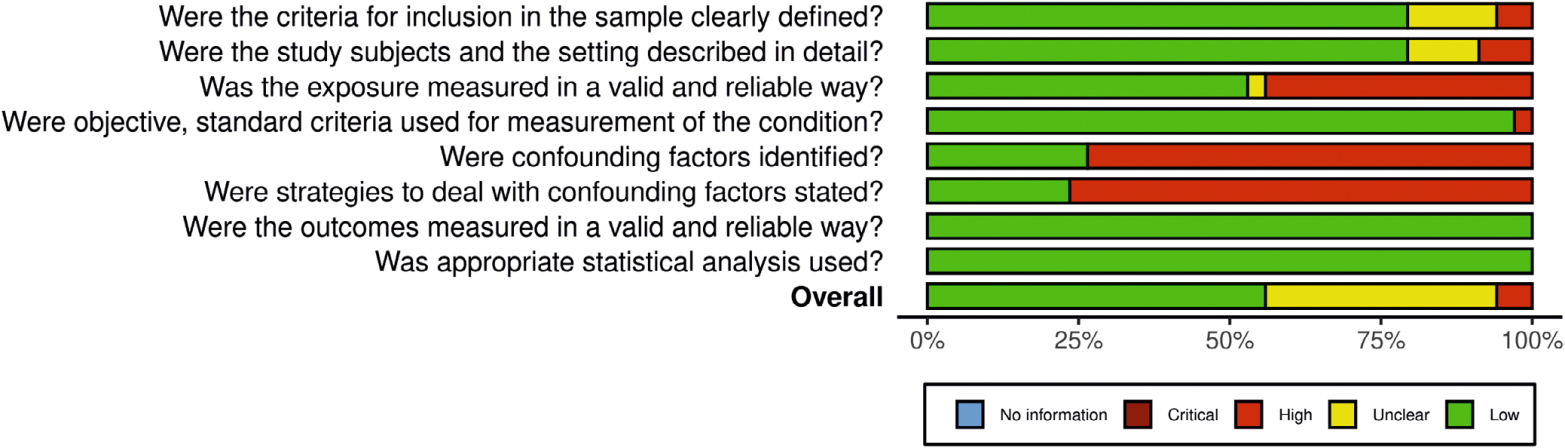
Weighted bar plots of the distribution of risk‐of‐bias judgements within each bias domain.

The main causes leading to studies being classified as having a high risk of bias were related to confounding factors (identification and strategies to deal with confounding factors) and the lack of valid and reliable ways to measure exposure (immunosuppression) (Figure 3).

### 3.4 Specific assessment of TTV in several conditions

#### 3.4.1 Solid organ transplant (SOT)

Analysis of the results from the 14 studies on TTV in SOT patients, which addressed those who received kidney, liver, lung and heart transplants, showed that the viral load was highly detectable even before the transplant. Although most authors have demonstrated that TTV can be a biomarker of immunosuppression in SOT patients^2^, ^4^, ^17^, ^30^, ^33^, ^34^, ^45^, ^50^ five studies showed that the viral load of TTV does not reflect the state of immune suppression^37^, ^46^, ^51^, ^52^, ^53^ and one study presented conflicting results, showing some parameters as positive and others as negative in the assessment of this association.^42^

Except for the studies conducted by Nordén et al.^37^ and Uhl et al.^42^ all other studies quantified the TTV viral load before and after the immunosuppressive events. They identified an increase in the quantity of the virus, particularly within the first 90 days after transplantation, when the viral load increased from a value between 3 and 4 log10 (pre‐transplant) to 6 to 9.5 log10.^2^, ^17^, ^30^, ^34^, ^45^, ^46^, ^50^, ^51^, ^52^, ^53^

It is important to note that only three studies evaluated immunological parameters,^2^, ^46^, ^51^ while the others assumed that the presence of infection^2^, ^17^ or the initiation of immunosuppressive medications was indicative of altered immune responses.^4^, ^30^, ^34^, ^45^, ^50^

#### 3.4.2 Hematopoietic stem cell transplantation (HSCT)

In 11 studies conducted on patients undergoing HSCT, it was possible to observe, in general, some similarities with studies on SOT, such as a high rate of positivity for TTV before transplantation^27^, ^31^, ^32^, ^39^, ^40^, ^41^, ^48^ and an increase in both positivity and viral load after transplantation.^31^, ^32^, ^40^, ^47^, ^48^

Although most studies reported an important difference between the control and transplant groups regarding TTV‐DNA levels, authors such as Peker et al.^39^ and Wohlfarth et al.^31^ considered this correlation statistically weak, in addition to reporting instability in relation to the viral load over time.^31^, ^39^ Indeed, Mouton et al.^27^ observed gradual post‐transplantation immune reconstitution, which could explain these changes in viral loads.^27^

The viral load of these patients, when compared to SOT patients, seems to present a wider range and reach higher levels both pre‐transplant (ranging from 2.4 to 9.36 log10) and post‐transplant (ranging from 6.4 to 10.15 log10), with the peak of TTV viral load in the post‐transplant period occurring between day +60 and +100.^35^, ^36^, ^40^

The efficacy of TTV as a biomarker of immunity was evaluated through various parameters.

Gilles et al.^36^ found that patients at lower risk of developing acute GVHD had higher leukocyte counts, lower infection rates and lower TTV viral loads than patients at a higher risk of developing acute GVHD (aGVHD).

Several studies have confirmed an inverse correlation between TTV viral load and lymphocyte count^31^, ^40^ as well as T‐cell function.^27^ Additionally, a positive correlation has been observed between the TTV viral load and the presence of other viruses,^31^, ^48^ as well as the onset of acute GVHD (aGVHD).^48^ The authors suggested that post‐transplantation complications and/or immunosuppressive drugs may affect TTV replication kinetics.^36^, ^48^, ^54^ However, among the evaluated immunosuppressive drugs, only melphalan and antithymocyte globulin (ATG) were associated with an increase in the TTV viral load.^32^, ^41^ Some studies have also found associations between the TTV viral load and patient survival and prognosis. For instance, Pradier et al.^40^ reported an overall survival rate of 50% in patients with a higher TTV viral load compared to 82% in those with a lower TTV viral load. Similar results were observed for progression‐free survival, with rates of 43% and 70% in patients with higher and lower TTV viral loads, respectively.^40^

On the other hand, some studies did not identify an association between the TTV count and the parameters described previously.^32^, ^39^, ^41^, ^47^ Zanotta et al.^35^ evaluated the cytokine profile after HSCT. IFN‐γ, TNF‐α, fibroblast growth factor‐basic (FGF‐basic) and monocyte chemotactic protein‐3 (MCP‐3) were found up‐regulated and significantly associated with TTV.

According to Wohlfarth et al.^31^ several variables can be correlated with TTV levels, but their complex interactions might perturb the capability of TTV to predict immune‐related complications.

#### 3.4.3 HIV+

Six studies were conducted to identify whether TTV could be a potential biomarker of immune status in HIV+ patients. Although all of them evaluated T CD4+ lymphocytes, TTV viral load was inversely correlated with CD4+ in three studies^13^, ^28^, ^44^ but was not associated with CD4+ count in the other three.^8^, ^26^, ^49^

The majority have demonstrated a higher detection of TTV viral load in HIV‐positive patients than in control patients.^13^, ^26^ For instance, Shibayama et al.^13^ and Madsen et al.^8^ demonstrated that TTV DNA was detected in 99% and 100% of the HIV+ treatment‐naive patients and in 91% and 42% of blood donor controls, respectively. Shieh et al.^26^ also identified a significantly higher detection rate of TTV in HIV+ patients than in controls, but the virus prevalence was much lower than that found in previous studies in both groups (32% and 16%, respectively).

In addition, Honorato et al.^28^ reported an association between TTV and patients with both AIDS and Kaposi's sarcoma. In these cases, TTV was detectable in the saliva of 87% of patients.^28^ Madsen et al.^8^ suggested that highly active antiretroviral therapy (HAART) allows better immune responses, even though patients do not present an increase in CD4+ T cells, suggesting that monitoring the TTV viral load could help in the evaluation of the cellular immune response in HIV‐positive patients receiving HAART.^8^

#### 3.4.4 Malignant neoplasias

TTV was detected in 69.8% of patients with melanoma before the immunotherapy treatment. However, there was no statistically significant difference between treatment‐susceptible and treatment‐resistant patients in terms of the mean values of TTV viral loads. There was also no statistically significant difference between the median value of TTV viral load over time, indicating that treatment has no direct impact on TTV replication, regardless of whether the tumor is sensitive or resistant to treatment.^43^

Sawata et al.^38^ carried out their study on patients with lung cancer and observed that the TTV viral load of 5.3 × 10^4^ copies/mL before chemotherapy decreased, on average, to 1.6 × 10^4^ copies/mL after treatment. However, when only patients with disease progression were considered, the mean TTV viral load significantly increased from 1.2 × 10^4^ copies/mL to 7.3 × 10^4^ copies/mL. In patients without idiopathic pulmonary fibrosis (IPF), it was observed that there was a decrease in the mean viral load of TTV was observed after chemotherapy, whereas patients with IPF had a higher mean viral load even after such a treatment.^38^

#### 3.4.5 Other diseases/conditions

Some researchers have also investigated the viability of TTV as a biomarker in other immunosuppressive conditions. Moen et al.^33^ evaluated the changes in TTV viral load in two groups of patients: soldiers undergoing extreme training known to cause immunosuppression and hepatitis C virus (HCV+) patients on interferon/ribavirin treatment. Falabello de Luca et al.^29^ verified whether the TTV viral load in cirrhotic patients on the transplant waiting list could be correlated with the count of circulating white blood cells. Both studies failed to demonstrate that TTV was a valid marker of immunity in the studied groups.

## 4 DISCUSSION

The management of immunosuppressed patients is directly linked to the degree of immune deficiency and the consequent possibility of opportunistic infections and malignant neoplasms. Being able to identify the level of the immune response, it is possible to try to prevent potentially fatal conditions that will certainly increase morbidity and decrease the patient's quality of life.

The diagnosis and assessment of the degree of immunosuppression depend on the clinical condition of the patient. It is well established that the CD4+ T lymphocyte count is the gold standard for evaluating the HIV+ patient's immune condition.^57^ In transplant patients, several biomarkers have been investigated to monitor immunosuppression in urine (perforin, granzyme B, CXCL9, CXCL10, CXCR3, or CD3E, or mRNAs) and in blood (donor‐specific antibody functionality, TRIB1, FOXP3, kSORT, miR‐142‐5p, T cell subgroups, IFNy‐ELISpot, B cell‐related genes, among others), but none of them have been validated so far. Among the potential methods to determine the immune function and the degree of immunosuppression, QuantiFERON Monitor®, ImmuKnow® and virus‐specific T cells (Tvis) are assays for whole blood which are currently under evaluation.^55^, ^58^, ^59^, ^60^, ^61^ There has also been an attempt to use pharmacological monitoring by measuring the levels of immunosuppressive drugs, but there is still no assay available for routine use.^55^, ^58^, ^59^, ^60^, ^61^ On the other hand, for other diseases or chronic systemic conditions that limit the individual's immune response but cause less overall impairment, there are no tests or laboratory parameters that have been developed or are being studied.^29^

Within the scope of new technologies that have been investigated to identify the degree of immunosuppression, TTV has been proposed as a potential endogenous biomarker for immune function. This possibility is based on the fact that the virus is universally widespread and highly prevalent in the global population, being present chronically in almost 90% of individuals, regardless of age, socioeconomic status, gender and health conditions.^62^ The fact that some TTV‐negative patients become TTV‐positive after immunosuppression suggests that the virus may persist in the body at low viral loads, which cannot be identified because of polymerase chain reaction (PCR) sensitivity or the transient absence of replication.^18^ Furthermore, the virus can be detected by PCR in different tissues and fluids (secretions and excretions), which facilitates the collection of samples. These facts, together with the low cost and speed of results (2 or 3 h, at most), make TTV quantification by PCR a promising candidate for evaluating any type of immunosuppression, unlike other methods restricted to transplanted patients.^62^

To confirm whether TTV can be a good candidate as an immunological biomarker, it is also necessary to verify whether the literature demonstrates the existence of a positive correlation between an increase in TTV viral load and states of immunosuppression.

A total of 3045 patients were evaluated in the 33 selected studies. Twelve studies (36.36%) did not find a correlation between TTV viral load and different types of immunological parameters. Four of the studies reported conflicting results. One study evaluated three different groups of patients, with only one of these groups (SOT) showing positive results. However, all studies found changes in TTV viral load in the context of an immunosuppressive event or a higher TTV viral load in the study group than in controls (although some were not statistically significant).

Although the risk of bias assessment (Figure 2) was classified as low in most studies (57.57%), it was possible to identify important clinical and methodological differences, making it unfeasible to perform a meta‐analysis.^56^ Even if we could divide the studies according to the patients' illnesses, there were differences in the study design (cross‐sectional and longitudinal), imbalances in the baseline population characteristics, differences in sample size (48.1% of the studies evaluated less than 50 patients), age (adults and children), diagnostic methods, fluid evaluated, immune parameters evaluated, adoption of a control group (55.55% had no control group) and different control group populations.

Solid organ transplantation (*n* = 14, 42.42%), HSCT (*n* = 10, 30.30%), HIV/AIDS (*n* = 6, 18.18%) and malignant neoplasms (*n* = 2; 6.06%) were the four main groups with different baseline immunosuppressive conditions. In addition to the intergroup biological differences in the aetiology of immunosuppression, there were intragroup variations in the types of treatment (drugs used in transplant and cancer; use of HAART or not), stages of the disease (e.g. AIDS, acute HIV infection and chronic HIV infection), presence of co‐infections (CMV, EBV, HBV, HCV, HGV, HHV‐8, HTLV‐1 and HPB‐19), sample collection intervals and outcomes.

Different parameters (CD4+ count, different cytokines and chemokines, presence of tumors and opportunistic infections, GVHD, presence of other viruses, acute and chronic graft rejection, lymphocyte and leukocyte levels, glomerular filtration rate and doses of immunosuppressive medications) were used to identify the presence of immunosuppression by comparing them with the TTV viral load.

We believe that such differences in methodologies did not invalidate the individual results of the studies, as almost all (except five) were performed to some extent longitudinal evaluation, which allowed for sequential monitoring of TTV viral load over time. Such information is important in this context. Viruses tend to have a fluctuating rate of replication which has not been set a threshold to interpret results at a single point in time in an individual patient. Therefore, so far, the evaluation of TTV is individualized for each patient and depends on the initial viral load that the patient presented.^63^ As positivity for the virus is common and viral load seems to fluctuate during a person's life,^64^ it is important to compare viremia at different times during a patient's follow‐up to assess immunosuppression states.

There is also no differentiation between what would be a ‘high’ or ‘low’ TTV viral load. The evaluations are individualized and carried out comparatively over a given period of time in the same patient or control group. To the best of our knowledge, only four studies have quantified and verified the fluctuation of the viral load in healthy individuals. Two studies investigated the dynamics of viral load in children and found that newborns presented viral load variations from 3.34 to 4.92 log10 copies/mL in the first weeks of life^65^ and from 0 to 5.99 log10 copies/mL in the first year of life.^64^ The other two studies evaluated adults, one of which quantified the TTV viral load in a single sample from a population of 512 adults' elite athletes, with a median of 6.43 log10 copies/mL, reaching a maximum of 11 log10 copies/mL.^66^ In the study by Focosi et al., 1017 healthy blood donors were evaluated and had a median of 2.3 log10 copies/mL (range 1.1 to 4.9 log10 copies/mL). Forty‐six were reevaluated 2 years later, and the TTV viral load remained essentially stable in the two samples.^67^

Indeed, in the future, it will be important to conduct studies comparing groups of patients exposed and not exposed to immunosuppression, collecting an equal number of samples from both groups at the same time points over the same period of time. This can provide important information in the context of performing clinical validity studies, also called diagnostic accuracy studies, with the aim of identifying a new diagnostic test to stage the level of immunosuppression in patients, as, in most conditions, there is no reference (gold) standard of diagnosis.^68^

The development of a commercial assay (TTV R‐GENE®) was one of the recently welcomed measures to standardize the methodologies in future studies, although most of the TTV primers targeted a highly conserved untranslated region in the TTV genome.^69^ Multicenter prospective trials with fixed time points from various immunosuppressive conditions and standardization of cutoff units are needed.^63^

Taking all of this into account, it is perhaps still unclear whether TTV monitoring can be used to assess the immunosuppression status of patients with different conditions, but there seem to be interesting indications regarding the ability to monitor the virus in transplanted patients.

This is the third systematic review of literature conducted about TTV. The previous systematic reviews sought to identify TTV as a marker of infection and rejection in SOT and in kidney transplantation. They also concluded that the evidence is weak^70^ and the studies presented substantial risk of bias.^69^

Further research with larger cohorts and controls over a longer period of follow‐up and standardized parameters will bring more certainty about TTV monitoring for different types of immunosuppression.

## 5 CONCLUSION

Most of the studies can identify the variation in TTV viral load in immunosuppressed patients, and some indicate a correlation between viral load and the individual's immunosuppression status. However, this does not guarantee a cause–effect relationship as there are many confounding factors. Further studies are required to confirm the use of TTV as an immune biomarker.

## AUTHOR CONTRIBUTIONS

FFVS and JBM conducted data analysis, interpreted results and wrote the report. BOR, RAVC and APJ were responsible for conducting the search, screening potentially eligible studies, extracting and analyzing data, updating reference lists and creating tables. KLO and PHBS contributed to designing the review protocol, conducting the search, screening potentially eligible studies and extracting data. MEPI and MPS were responsible for designing the review protocol and providing feedback on the report.

## FUNDING INFORMATION

Fundação de Amparo a Pesquisa do Estado de São Paulo—FAPESP (grant number: 2021/07490‐0) and Pró‐Reitoria de Pesquisa, Universidade de São Paulo (grant number: 2021.1.10424.1.9).

## CONFLICT OF INTEREST STATEMENT

The authors declare that they have NO affiliations with or involvement in any organization or entity with any financial interest in the subject matter or materials discussed in this manuscript.

## Supporting information


Appendix S1.

## Data Availability

The authors confirm that the data supporting the findings of this study are available within the article and its Appendix S1.
