# Supplementary material for: Torque teno virus as a marker of immune status in immunocompromised patients: A systematic review
Source: Eur J Clin Invest. 2025 May 15;55(8):e70068. doi: 10.1111/eci.70068 (PMC12257264; doi:10.1111/eci.70068)
Supplement: Supplementary file 1 — Appendix S1. [file ECI-55-e70068-s001.docx]

Therapeutic regimens in solid organ transplant recipients

| **Author, year, country** | **Organ** | **Immunosuppressive regimen (IT)**  **- induction** | **Immunosuppressive regimen (IT)**  **- maintenance** | **Treatment for rejection** | **Correlation betweeen TTV VL and IT** |
| --- | --- | --- | --- | --- | --- |
| Moen *et al.*, 2003;  Norway | Kidney | Basiliximab or azathioprin or MMF | Standard doses of CSA, and prednisolon. Four patients received tacrolimus instead of CSA. | Ten standard doses of  OKT3 antibodies or two standard  doses ATG | NA |
| Focosi *et al.*, 2015;  Italy | Kidney and/or Pancreas | Anti-T-cell agent (either  ATG days 0–6 (n523) or basiliximab 20 mg days 0–4 (n547)) and methylprednisolone 500 mg. Six patients received ATG 0.8 mg kg21 day21, while the remaining 17 received a dose of 1 mg kg21 day21.  The authors did not present the data separated by the type of transplanted organ | Tacrolimus, MMF and prednisone | NA | There was no statistically significant difference in TTV viraemia at any time point between patients receiving ATG 0.8 mg kg21 day21 versus 1 mg kg21 day21. |
| Maggi *et al.*, 2018;  Italy | Kidney or Liver | ATG-based or basiliximab-based  The authors did not present the data separated by the type of transplanted organ | Regimen based on CSA and regimen based on tacrolimus | NA | ATG and CSA based-regimens presented higher median levels of TTV when compared to basiliximab and tacrolimus-based regimens, respectivelly.  Mean TTV levels did not difer when compared to tacrolimus doses within 5–15ng/ml or beyond the therapeutic ranges (<5 and >15ng/ml) and viral loads and drug levels were not found to correlate at any time post-transplant |
| Fernández-Ruiz *et al.*, 2019; Spain | Kidney | ATG or brasiliximab or none | NA | NA | ATG was associated with higher TTV viral load through the first 6 posttransplant months |
| Uhl *et al.*, 2020;  Austria | Kidney-children | NA | Corticosteroids, MMF or azathioprine, and calcineurin inhibitors or rapamycin | NA | TTV load positively correlated with the dose of prednisolone and MMF |
| Batista *et al.,* 2022;  Brazil | Kidney | NA | Tacrolimus + Sodium  mycophenolate +  Prednisone  Tacrolimus + Azathioprine +  Prednisone  Tacrolimus + Everolimus +  Prednisone  CSA + Azathioprine  + Prednisone | NA | A higher viral load in plasma was observed for a drug regimen consisting of tacrolimus, sodium mycophenolate and prednisone compared to other regimens |
| Benning *et al*., 2023; Germany | Kidney | NA | CNI + MPA + CS and 8 patients with immunosuppression other than CNI + MPA + CS | NA | No significant differences in TTV load were  seen between kidney transplant recipients (KTRs) who continued immunosuppressive  maintenance therapy including mycophenolic acid compared with KTRs who withdrew |
| Querido *et al*., 2023; Portugal | Kidney | Basiliximab (20 mg IV) or ATG (1.25 mg/kg/day IV).  Since the first day until the  seventh day after kidney transplant. Methylprednisolone (500 mg on 1st day, 250 mg on 2nd day, 125 mg on 3rd, and 80 mg on 4th day IV after kidney transplant) was included in all protocols. Rituximab (375 mg/m2 for 2 doses 2 weeks apart) were used as induction therapy in addition to thymoglobulin in two highly sensitized patients. | Tacrolimus, MMF, and prednisone. Tacrolimus was administered orally at 0.15 mg/kg/day divided in two doses and adjusted to maintain a target trough concentration between 4 and 10 ng/mL, depending on the time elapsed after kidney transplant. Prednisolone was prescribed since the fifth day after KT (0.6 mg/kg) and was tapered to 5 mg/day during the first 3 months after kidney transplant. MMF (1000 mg orally twice daily) was started after kidney transplant and was reduced if adverse events appeared, otherwise it was reduced to 1000 to 1500 mg daily dose after the first 3–6 months. | NA | NA |
| Mafi *et al*., 2023; France | Kidney | Basiliximab or thymoglobulin | Tacrolimus or CSA combined with IMPDH inhibitors and corticosteroids | NA | NA |
| Cañamero *et al*., 2023; Spain | Kidney | Basiliximab or thymoglobulin | Twice daily tacrolimus, MMF, and prednisone | NA | TTV loads at month 1 and at month 3 were  not associated with thymoglobulin or any tacrolimus level |
| Berg *et al*., 2023; Denmark | Heart | anti‐thymocyt globulin for the first 3 postoperative days | Tacrolimus and methylprednisolone for the first 2 postoperative days followed by prednisolone (0.3 mg/kg/day), which was gradually reduced during the first postoperative year | NA | No correlation between TTV viral load and serum levels of tacrolimus |
| Görzer *et al*, 2015;  Austria | Lung | A single dose of Alemtuzumab, 30 mg | Tacrolimus target trough levels were 10–12 ng/mL within the first three post-operative months. Prednisolone was started on day one post-operatively at 1 mg/kg/day, and then tapered to 0.2 mg/kg/day within the first three months.  Mean tacrolimus target trough levels ranged between 7.8–14.0 ng/mL during the first 30 days, and between 8.4–14.0 ng/mL during 60 days post-transplantation | NA | No significant correlation between TTV viral load and tacrolimus blood drug level was observed for 30 and 60 days. |
| Nordén *et al.*, 2018;  Sweden | Lung | ATG,  which was given for 1–3 consecutive days together with methyl-  prednisolone intravenously. | Prednisone 0.3 mg/kg/d and MMF 2 g/d. The patients then received either oral  CSA; 1–2 mg/kg, adjusted to maintain a serum level of 300–350 ng/mL, or tacrolimus (TAC; 0.075 mg/kg)  given orally divided in 2 doses daily, adjusted to maintain a serum level of 14–16 ng/mL. | NA | CSA-treated patients had significantly lower TTV-DNA levels in serum at month 6 post-LTx and onwards, compared with the tacrolimus-treated patients |
| Jaksch *et al.*, 2018;  Austria | Lung | Alemtuzumab or  ATG or no induction therapy | Patients who received alemtuzumab were on dual therapy with tacrolimus (target level 8-10 ng/ml first 3 months, 6-8 ng/ml months 4-12) and steroids within the first post-transplant year, MMF was added after 12 months (1-2g/day). | NA | A small but statistically significant correlation of alphatorquevirus and tacrolimus levels |

*Abreviations:* TTV, torque teno virus; VL, viral load; IT, immunosuppressive regimen; NA, not available; ATG, anti-thymocyte globulin; BU, busulfan; CSA, ciclosporin; CY, cyclophosphamide; FBM or BEAM, fludarabine, carmustine (BCNU), melphalan; FLAMSA, fludarabine, cytarabine, amsacrine; FLU, fludarabine; GVHD, graft-versus-host disease; MTX, methotrexate; TBI, total body irradiation; TREO, treosulfan; MMF, mycophenolate mofetil; G-CSF, granulocyte colony-stimulating fator;

Therapeutic regimens in Hematopietic stem cell transplantation patients

|  | **Conditioning regimen** | **GVHD profilaxis** | **Correlation betweeen TTV VL and therapeutic regimens** |
| --- | --- | --- | --- |
| Focosi *et al.*, 2010;  Italy | Multiple myeloma - Melphalan (70–200 mg/m2) on day −2 relative to the  transplant and G-CSF 10 mcg/kg on day +1;  Lymphoma - BEAM polychemotherapy (BCNU 300 mg/m2 on day −7, etoposide 100 mg/m2 and ara-C 200 mg/m2 on days −6 to −3, melphalan 140 mg/m2 on day −2) and G-CSF on day +1. | NA | NA |
| Zanotta *et al.*, 2015;  Italy | NA | NA | NA |
| Gilles *et al.*, 2017;  Germany | FLU/BU/ATG  FLAMSA/TBI/ATG  FLU/BU  FLU/BU/CY  FLU/BU/CY  FBM/ATG  BU/CY/ATG  TLI/ATG  BU/CY  TBI/FLU/ATG  FLAMSA/TBI  TBI/FLU/RITUXI-  MAB/ATG | CSA in combination with MTX or with MMF | NA |
| Wohlfarth *et al.*, 2018;  Austria | Myeloablative  Reduced intensity  Non-myeloablative | CSA + MTX after  myeloablative conditioning, and CSA + MMF following  reduced-intensity or non-myeloablative conditioning.  In addition, 46% patients received ATG. | NA |
| Mouton *et al.*, 2020;  France | Myeloablative  Reduced intensity  TBI | Antithymocyte globulins, cyclosporine,  tacrolimus, methotrexate, mycophenolate mofetil, cyclophosphamide, and corticosteroids | **NA** |
| Peker *et al.*, 2020;  Turkey | Myeloablative  Non-myeloablative | CSA in combination  ATG, MTX, MMF and/or corticosteroids | There was no significant difference between GvHD prophylaxis regimens and TTV-DNA loads |
| Pradier *et al.*, 2020;  Switzerland | Myeloablative - CY 120 mg/kg in combination with  TBI (10–12 Gy) or BU 12.8 mg/kg intravenously.  Reduced intensity - fludarabine 150 mg/m2, associated with BU 6.4 mg/kg intravenously or melphalan 140 mg/m2 and ATG | CSA in combination with either MTX, in case of MAC, or MMF for patients transplanted after reduced intensity conditioning  T cell depletion (TCD) consisted of administration of ATG and/or “in vitro” partial T cell depletion of grafts. Partial T cell depletion (pTCD) graft recipients also received methylprednisolone on days −2 and −1. Patients receiving grafts from haploidentical donors received CY (50 mg/kg) on days 3 and 4 post-HSCT | TTV kinetics in patients receiving TCD were similar to those in no TCD group.  only patients receiving ATG together with pTCD grafts exhibited TTV titers higher than no TCD patients |
| Schimitz *et al.*, 2020;  Germany | Myeloablative - BU > 12mg/kg body  weight or melphalan 200 mg/m2 alone or together with TBI  Reduced intensity - reduction of at least 30% in cytostatic and irradiation intensity.  Eighty-seven patients received ATG | MMF + CSA or MMF + tacrolimus | a higher ATG dose was associated with a significantly higher TT viral  load.  There was a clear but no significant trend towards higher TT viral loads within the group that received tacrolimus as GvHD prophylaxis.  No significant differences in TTV viral load depending on the drug concentration were found.  Patients with myeloablative regimen (n= 48) showed significantly higher TT viral loads than patients with reduced intensity conditioning regimen. |
| Spiertz *et al*., 2023; Germany | Myeloablative  Reduced intensity | CSA+MTX+MMF | NA |
| Forqué *et al*., 2023; Spain | Myeloablative  Reduced intensity | Sirolimus + MMF + CY  Tacrolimus + sirolimus  Tacrolimus + CY | NA |

*Abreviations:* TTV, torque teno virus; VL, viral load; IT, immunosuppressive regimen; CsA, cyclosporin; NA, not available; ATG, anti-thymocyte globulin; BU, busulfan; CSA, ciclosporin; CY, cyclophosphamide; FBM or BEAM, fludarabine, carmustine (BCNU), melphalan; FLAMSA, fludarabine, cytarabine, amsacrine; FLU, fludarabine; GVHD, graft-versus-host disease; MTX, methotrexate; TBI, total body irradiation; TREO, treosulfan; MMF, mycophenolate mofetil; G-CSF, granulocyte colony-stimulating fator.
